# Establishment of an enhanced recovery after surgery protocol in minimally invasive heart valve surgery

**DOI:** 10.1371/journal.pone.0231378

**Published:** 2020-04-09

**Authors:** Jens C. Kubitz, Leonie Schulte-Uentrop, Christian Zoellner, Melanie Lemke, Aurelie Messner-Schmitt, Daniel Kalbacher, Björn Sill, Hermann Reichenspurner, Benedikt Koell, Evaldas Girdauskas

**Affiliations:** 1 Department of Anaesthesiology, University Medical Center Eppendorf, Hamburg, Germany; 2 Department of Physiotherapy, University Medical Center Eppendorf, Hamburg, Germany; 3 Department of General and Interventional Cardiology, University Heart Center Hamburg, Hamburg, Germany; 4 Department of Cardiovascular Surgery, University Heart Center Hamburg, Hamburg, Germany; Universita degli Studi di Roma La Sapienza, ITALY

## Abstract

Protocols for “*Enhanced recovery after surgery* (ERAS)” are on the rise in different surgical disciplines and represent one of the most important recent advancements in perioperative medical care. In cardiac surgery, only few ERAS protocols have been described in the past. At University Heart Center Hamburg, Germany, we invented an ERAS protocol for patients undergoing minimally invasive cardiac valve surgery. In this retrospective single center study, we aimed to describe the implementation of our ERAS program and to evaluate the results of the first 50 consecutive patients. Our ERAS protocol was developed according to a modified Kern cycle by an expert group, literature search, protocol creation and pilot implementation in the clinical practice. Data of the first 50 consecutive patients undergoing minimally invasive cardiac valve surgery were analysed retrospectively. The key features of our multidisciplinary ERAS protocol are physiotherapeutic prehabilitation, minimally invasive valve surgery techniques, modified cardiopulmonary bypass management, fast-track anaesthesia with on- table extubation and early mobilisation. A total of 50 consecutive patients (mean age of 51.9±11.9 years, mean STS score of 0.6±0.3) underwent minimally-invasive mitral or aortic valve surgery. The adherence to the ERAS protocol was high and neither protocol related complications nor in-hospital mortality occurred. 12% of the patients developed postoperative atrial fibrillation, postoperative delirium emerged in two patients and reintubation was required in one patient. Intensive care unit stay was 14.0±7.4 hours and total hospital stay 6.2±2.9 days. Our ERAS protocol is feasible and safe in minimally-invasive cardiac surgery setting and has a clear potential to improve patients outcome.

## Introduction

One of the most important improvements in contemporary perioperative medicine has been the establishment of *enhanced recovery after surgery* (ERAS) protocols in surgery [1]. In general surgery, especially colorectal surgery [2, 3], major advantages in terms of gastrointestinal morbidity [4], health-care associated infections [5] and earlier hospital discharge have been demonstrated for such protocols. An ERAS protocol is a multidisciplinary and multiprofessional effort including: (1) an improvement of the preoperative physical and mental status (e.g., exercising the cardio-pulmonary capacity and optimization of the nutritional status) (2) a modification of the intraoperative management by standardized minimally-invasive surgery and an early extubation, and finally (3) an implementation of enhanced postoperative recovery protocol integrating an individualized analgesia and standardized physiotherapeutic program. Those are the key features of an integrative ERAS protocol which extends beyond the intraoperative phase. Major success of such protocols led to the establishment of an international ERAS Society and the initiation of ERAS protocols in the adjacent surgical disciplines, closely associated with the colorectal surgery. So far, the ERAS society has developed protocols for pancreatic surgery [6, 7], bariatrics [8], urology [9] and gynaecology/ obstetrics [10] which all have been certified by the ERAS Society.

For cardiac surgery, so called fast-track protocols have been published in the past [11]. They demonstrated, that an early extubation and faster recovery after cardiac surgery is safe and feasible and that the post-anaesthetic care or ICU stay can be significantly shortened by implementing such fast-track protocols [12]. However, the majority of the so far published ERAS protocols in minimally-invasive cardiac surgery did not consider the complete perioperative process, as described above. ERAS protocols in the cardiac surgery are still in a premature stage. A recently published study described a protocol for ultra-fast track minimally invasive aortic valve replacement and included some of the characteristics of the ERAS protocol [13]. In support of the mission and efforts of the ERAS society to spread the idea of ERAS to further disciplines [14] and to create a protocol for minimally invasive valve surgery (i.e., aortic and mitral valve surgery), this single-center pilot project including 50 consecutive patients undergoing minimally-invasive cardiac surgery was performed. Primary endpoint of our study was to demonstrate the feasibility and safety of our novel ERAS protocol.

## Methods

### Study design and ethical approval

This is a retrospective single-center study performed at the University Heart Center Hamburg, Germany. It is in accordance with the Declaration of Helsinki, released 2008. The first fifty patients who had entered the institutional ERAS conform protocol for minimally invasive cardiac surgery from first of February to first of July 2018 were retrospectively analysed (Approval by our local Ethics Committee, Ethikkommission Hamburg, PV7050). As this is a retrospective study, written informed consent was not required according to the Ethics Committee. Nonetheless, all patients that are admitted to the University Heart and Vascular Center Hamburg are systematically asked for their consent to use their anonymized health- and treatment-related data in ongoing research project. All patients included in this study gave their general consent to analyse their data retrospectively.

Inclusion criteria for this study were isolated minimally invasive aortic or mitral valve surgery and age < 70 years. Exclusion criteria were concomitant coronary artery or aortic valve disease requiring simultaneous surgery through a complete sternotomy, redo surgical procedures, patients unwilling to participate at ERAS program and patients with severe comorbidities (i.e., prior stroke or terminal renal insufficiency) unsuitable for ERAS due to interdisciplinary team decision after preoperative consultation.

The patients were screened systematically from a pool of referrals for minimally invasive heart valve surgery at our center. All patients were contacted by phone and asked to undergo preoperative consultation at the out-patient unit of our center. Only those patients who were willing to participate at the ERAS program were included in this study.

### Development of the program

**Fig 1:**
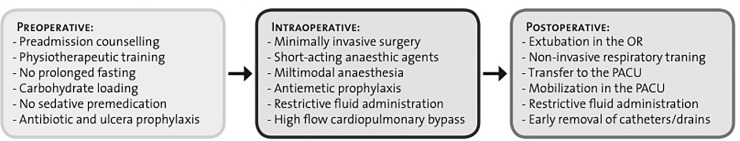
Key features of the ERAS program (in accordance with the recommendations of the Enhanced Recovery after Surgery Society®).

Our ERAS protocol for cardiac surgery was developed according to a simplified Kern cycle [15]. The first step was a requirement analysis based on a literature review, as described in the introduction, and an interdisciplinary and interprofessional expert group analysis at our institution. This expert group consisted of one cardiac surgeon, one anesthetist, one perfusionist and one physiotherapist. The second step was the description of an integrative ERAS protocol with its key features displayed in Fig 1. The protocol is in accordance with the recent Guidelines for Perioperative Care in Cardiac Surgery Enhanced Recovery After Surgery Society Recommendations [14]. The third step was the pilot implementation of the ERAS protocol at our institution. The fourth and final step was the evaluation of the first 50 consecutive patients, as presented in the results section of the manuscript.

### Prehabilitation

All included patients are invited to attend a preoperative interdisciplinary meeting with the surgeon, physiotherapist, nursing staff, psychologist and rehabilitation team two to three weeks before the scheduled surgery. All patients undergo formal assessment regarding their present physical condition (i.e., frailty scoring using LUCAS scale [16]) and their suitability/motivation to participate at the ERAS program. Furthermore, they are introduced into the program, especially into the physiotherapeutic exercises’ module. The patients are asked to perform daily exercising activities two to three weeks prior to surgery for prehabilitation as well as in order to be familiar with the exercises prior to the postoperative period. Further, the nutritional status and blood testing are checked and corrected, as indicated. Supplemental nutritional support is recommended for the last 2 weeks before the scheduled surgery by means of high-energy, high-carbohydrate diet (e.g. Impact Drink (Nestlé), Fresubin (Fresenius Kabi), Fortimel (Nutricia)).

On the evening prior to surgery and on the day of surgery, patients receive omeprazole 20 mg for prevention of gastric ulcers. Before admission to the OR, a carbohydrate drink (Maltose 25g) and oral premedication with midazolam 7.5 mg are administered.

### Intraoperative anesthesiologic management

On arrival in the OR, standard monitoring with an arterial line is applied and dexamethasone 4 mg (for antiemetic prophylaxis) and Ranitidin 50 mg (for additional prophylaxis of gastric ulcers) are administered. Anaesthesia is induced with Sufentanil 50 μg and Propofol 1.5 mg kg^-1^ and neuromuscular blocking is performed once with Rocuronium 0.6 mg kg^-1^. After intubation, a central venous line and a 7Fr. introducer sheath are regularly inserted in an internal jugular vein. All patients are monitored with transesophageal echocardiography.

Anaesthesia is maintained with Remifentanil (0.4–0.5 μg kg^-1^ min^-1^), Propofol (2mg kg^-1^ h^-1^) and a variable Sevoflurane concentration (end-tidal Vol. % 0.6–1.8). Depth of anesthesia is guided by bispectral index monitoring (target 40 to 50) throughout the procedure. On CPB, Sevoflurane is administered via the CPB circuit. No further neuromuscular blocking drug is administered. 15 min before end of surgery, 1g Metamizole and 7.5 mg Piritramid are administered for postoperative analgesia and 4 mg Ondansetron for prophylaxis of postoperative nausea. The additive continuous propofol infusion was implemented because the first 20 patients suffered a severe postoperative nausea/vomiting.

On cardiopulmonary bypass (CPB) mean arterial blood pressure of at least 50 mmHg is tolerated if pump flow is ≥ 3.2 l m^2^.min^-1^ and cerebral oximetry (NIRS) values are within 10% range of baseline value. If NIRS drops below 10% of baseline value, mean arterial pressure is increased with continuous vasopressor therapy (noradrenaline) to baseline value and FiO_2_ and/or haemoglobin are elevated if indicated. Transfusion trigger is haemoglobin < 4.5 mmol.l^-1^ (resp. 7,5g.dl^-1^). Restrictive fluid therapy is implemented during CPB with the goal of negative fluid balance at the end of the procedure. Crystalloid solutions (Sterofundin® ISO (B.Braun)) are preferentially used to compensate diuresis. Atrial fibrillation prophylaxis was started intraoperatively using a continuous amiodarone infusion (900mg.50ml^-1^) with a rate of 10ml.hour^-1^ for the first 24 hours in patients who had an enlarged left atrium, reduced left ventricular ejection fraction and previous history of atrial fibrillation.

For weaning from CPB, noradrenaline is administered to maintain a mean arterial pressure above 60 mmHg and epinephrine is added depending on patients needs assessed with transesophageal echocardiography.

### Perfusion management

The cardiopulmonary circuit is primed with crystalloid solution (Jonosteril 850 ml (Fresenius Kabi)), Mannitol 20% (100 ml) and 100ml of albumin 20%. Bypass flow is targeted to > 3.2 l m^2^.min^-1^ and core temperature is lowered to 32–33°C. For minimally-invasive valve surgery, we routinely implement crystalloid Custodiol cardioplegia (> 20–30 ml/kg (Dr. Franz Köhler Chemie)). While on CPB, routine hemofiltration is used for removal of the priming volume and cardioplegia targeting a zero to negative fluid balance. After aortic clamp release, a reperfusion period of 30% of the aortic clamp time is performed, prior to weaning from cardiopulmonary bypass. The patient is rewarmed to 37.5°C by means of the cardiopulmonary circuit and a heating blanket.

### Surgical management

In the pilot phase, we adopted our ERAS protocol for elective cardiac surgical patients scheduled for minimally-invasive aortic and mitral valve surgery.

**(1) Aortic valve surgery** with or without simultaneous aortic root/ ascending aortic surgery is performed using a partial upper T mini-sternotomy approach in the third intercostal space. Cannulation for CPB is established using a percutaneous right femoral vein drainage (Smart-Flow cannula by Smartcanula LLC) and distal ascending aortic inflow cannula (Medtronic). A left-sided vent is routinely inserted through the right superior pulmonary vein. Patients with an isolated/predominant aortic valve regurgitation undergo aortic valve repair procedures which consist of aortic valve annular stabilization and aortic cusp repair. Tissue valve prostheses are predominantly used in the remaining patients who present with a predominant aortic valve stenosis. Epicardial temporary pacing leads are routinely applied for atrial and/or ventricular pacing, as required. Single intrapericardial chest tube is inserted through a subxyphoidal access. Chest closure is performed with six sternal wires. Sterile vancomycin pasta (3g vancomycin) is routinely inserted between sternal halves before closure.

**(2) Mitral valve surgery** with or without concomitant tricuspid valve surgery, left atrial ablation, and closure of left atrial appendage is performed through a four to five cm right anterolateral incision in the fourth intercostal space. Perimammillar incision is routinely used in males. 3D full-endoscopic (Einstein Vision, Aesculap) non-rib spreading approach with a soft-tissue retractor is implemented and transthoracic aortic cross-clamp is introduced through the third intercostal space. CPB was established using a femoro-femoral cannulation in all patients without signs of systemic atherosclerosis as for patients with aortic valve surgery. Axillary artery inflow is used in all patients with an evidence of systemic atherosclerosis (e.g. calcifications in the downstream thoracic aorta, atherosclerotic plaques in the carotid or femoro-iliac vessels). Mitral valve repair was performed using standardized techniques for degenerative and functional mitral valve regurgitation. Epicardial temporary pacing leads are routinely sutured on the right atrium and ventricle for temporary postoperative pacing, as required. Single chest tube is placed in the right pleural cavity and local anesthetic agent (Ropivacain 0.75%, 10 ml) is injected in the fourth intercostal space. Furthermore, an intercostal catheter (CADD^®^-Solis (B Braun) is introduced in the forth intercostal space for regional anaesthesia after the surgery.

### Postoperative early recovery protocol

#### (A) Postoperative Anaesthesia Care Unit (PACU)

All patients are weaned from mechanical ventilation and extubated in the OR prior to their transfer to the PACU. In the first hour of PACU stay, chest x-ray and standard laboratory tests are conducted and short-term non-invasive mechanical ventilation is routinely performed for lung recruitment. Further, patients are regularly admonished to use a respiratory therapy device (YPSI Set Medisize, Finnland). Pain management strategy contains a fixed dose of Metamizole (1g) and Piritramid. In patients after lateral after minithoracotomy, continuous regional anaesthesia is administered with a fixed catheter infusion rate of 6 ml.h^-1^ ropivacain 0.2% until the first postoperative morning. Fluid balance is kept neutral to negative for the first 24 hours postoperatively. In the case of insufficient diuresis (<1ml kg^-1^.h^-1^), intravenous diuretics (Furosemide 10–20 mg) are administered. If patients suffer from PONV, administration of Ondansetron (4 mg) is repeated and, if necessary, additionally supplemented with Droperidol 0.625–1.25 mg.

All patients receive their first postoperative physiotherapy treatment in the PACU, two to three hours after surgery with a passive mobilization in bed, respiratory exercises and an active mobilization to the upright position. First postoperative transthoracic echocardiography is performed by a cardiologist prior to the transfer to the intensive care unit (ICU).

#### (B) Intensive Care Unit (ICU)

At this pilot stage of our ERAS project, all patients are transferred to ICU over the night after surgery for safety and monitoring reasons. The patients undergo their second physiotherapy session by ICU nurse personnel in the evening and have their first postoperative food intake in the sitting position. Chest tubes, invasive arterial line and central venous catheter are removed after 12 hours postoperatively. The patients are routinely transferred to the low care ward directly after morning ICU round. Fixed postoperative pain medication is maintained according to our ERAS protocol.

#### (C) Physiotherapy

*(1) Prehabilitation*. We introduce the patients into the physiotherapy protocol during the pre-operative consultation which includes teaching on the breathing coach and respiratory exercises to prevent post-operative atelectasis and pneumonia. The patients were encouraged to perform self-assessments to optimize their analgesic management, to positively influence the compliance and postoperative exercising. Patient-oriented pain management is deemed to facilitate intensive physiotherapy units during hospitalization. Furthermore, the patients were educated to maintain strength and endurance training for at least 2 weeks before the surgery, based on the preoperatively measured physical status (size, weight, handgrip strength, 1min-sit-to-stand), frailty index (LUCAS, Timed- up&go) and usual physical activity (occupation, sports). This prehabilitation was aimed to educate a well-informed and motivated patient with a high personal responsibility level. To monitor physiotherapeutic progress after the surgery, the responsible physiotherapist checks daily physical activity level using patient self-monitoring sheets.

*(2) Day of the surgery*. First physiotherapy unit is scheduled three hours after the surgery in the PACU. The decision to mobilize the patient is made in consensus with the responsible anaesthetist. During this monitor-guided mobilization, we followed the *break-off /stop criteria* according to the recommendations of S2 guideline of the German Society of Anaesthesiology and Intensive Care Medicine [17]. First, the physiotherapist checks motoric and sensory functions of upper and lower extremities in supine position as well as the postoperative pain level. If the pain level is low to moderate, the patient is encouraged to sit down at the edge of the bed. After performing intensive respiratory therapy in the sitting position, the mobilization is continued to the upright position beside the bed and making of the first steps.

*(3) First postoperative day*. Four physiotherapy units are routinely scheduled on the postoperative day one. The first unit is accomplished on the ICU. The patient walks with physiotherapist support on the floor. The three subsequent physiotherapy units are performed on the postoperative ward and aim to increase the walking distance. The patient is additionally instructed regarding the break management during physical activities and continues to perform breathing exercises. Physical activity and breathing exercise are documented in a physiotherapy chart.

*(4) Second postoperative day*. In case of an uneventful postoperative course (e.g. adequate sleep, food intake and low to moderate pain level), we additionally include stairs climbing and ergometer exercising with a moderate intensity during the second postoperative day. The patients are encouraged to document their physical activity and breathing exercises in the physiotherapy chart.

*(5) Third and fourth postoperative days*. The patients are encouraged to exercise independently during the postoperative days three and four. The physiotherapist monitors only the intensity of physical activities and breathing exercises and supports in case of any difficulties.

### Physiotherapy protocol adjustments

In the beginning, the very first 20 patients complained quite often about increased nausea/vomiting during the first and second postoperative days. This condition had a negative impact on the energy supply. Therefore, it was common to defer physiotherapy units to the third and fourth postoperative day. After combining continuous propofol and sevoflurane administration for maintenance of anaesthesia instead of sevoflurane administration alone, the patients felt much more comfortable and showed a much better compliance with the physiotherapy protocol (see also **Table 5**).

Some patients underestimated the importance of postoperative chest pain or ignored regular pain self-assessments which impeded the postoperative mobility. Consequently, we designed a checklist to better evaluate the compliance during the pre-operative counselling and developed a booklet addressing the most important aspects of ERAS physiotherapy program.

(D) Discharge to rehabilitation. The patients were managed postoperatively with the primary aim to discharge them home or to the rehabilitation facility on the forth to fifth postoperative day. Pacemaker wires and peripheral venous line were removed on the third postoperative day. Before discharge, transthoracic echocardiography and routine laboratory exams were performed. The decision to discharge was confirmed in a brief daily meeting of the interdisciplinary ERAS team.

### Implementation of the protocol and regular review

After developing the protocol as described above, all members of the specialties named above and all the nurses on the ward were made familiar with the protocol. For the first 20 patients, regular rounds in the PACU, the ICU and on the ward were held by the surgeon, the anaesthetist, the attending nurse and the physiotherapist. For interdisciplinary feedback and team building purposes, a monthly ERAS board meeting is organized to review each case and to discuss frequent problems. Exemplary, postoperative nausea and vomiting was a major issue at the beginning of the program and, after discussing at the ERAS board meeting, could be significantly reduced by the modifications descrived above.

### Statistical analysis

Statistical analysis was performed using SPSS Version 23.0 (IBM Corp, New York, USA). Categorical variables are presented as percentages and continuous variables are expressed as mean ± standard deviation (SD) throughout the manuscript. Continuous variables were compared using the unpaired two-sided t-test. Categorical variables were analyzed using the chi-square test or the Fisher’s exact test, as appropriate. Data were tested for normal distribution using the Kolmogorov–Smirnov test. P-values of < 0.05 were considered statistically significant.

## Results

**Fig 2:**
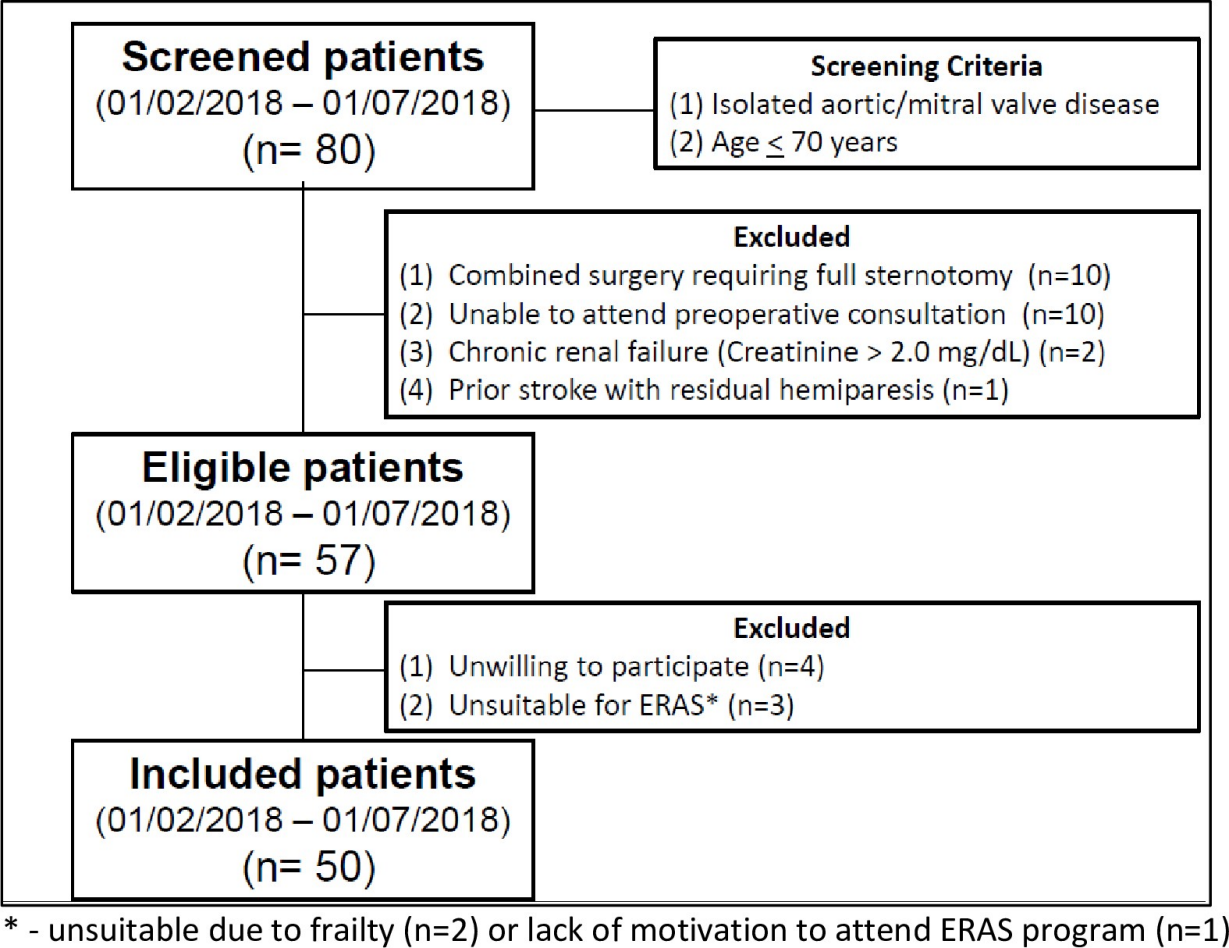
Screened, eligible and finally enrolled patients.

The above described ERAS protocol was implemented during a pilot phase in the first 50 consecutive patients undergoing a minimally invasive mitral or aortic valve surgery. **Fig 2** shows the flow-chart of the screened, eligible and finally enrolled patients.

Demographics and baseline characteristics of our pilot patient cohort are displayed in **Table 1**.

**Table 1:** Demographics and baseline variables. Values are presented as numbers (proportion) or as mean ± SD. CAD- coronary artery disease, NYHA–New York Heart Association functional class.

Variable	Study cohort (n = 50)
Mean age (years)	51.9 ± 11.9
Sex (male / female)	38 / 12
Body Mass Index (kg/m^2^)	26.1 ± 3.1
Surgical Risk scores:	
- STS score	0.6 ± 0.3
- Log. EuroScore	1.7 ± 0.9
Comorbidities	
- NYHA class	2.0 ± 0.8
- Hypertension	24 (48%)
- CAD	5 (10%)
- Atrial fibrillation	7 (14%)
- Smoking	6 (12%)
- Diabetes	2 (4%)

Briefly, our study cohort consisted of relatively young, low surgical risk patients that were scheduled for an elective minimally invasive aortic or mitral valve surgery. The most common comorbidity was arterial hypertension, which was present in 48% study patients. All patients were prepared for surgery according to the preoperative ERAS protocol, as described in Fig 1. The surgery was performed by five dedicated heart valve surgeons at our institution. The details of surgery and of postoperative management are summarized in the Table 2.

**Table 2:** Intraoperative and postoperative management. Values are presented as numbers (%) or as mean ± SD. CPB- cardiopulmonary bypass; ICU- intensive care unit; OR- operating room; PACU- post-anesthesia care unit.

Surgical variables	Study cohort (n = 50)
Mitral valve surgery	23 (46%)
- mitral valve repair	22 (44%)
- mitral valve replacement	1 (2%)
Aortic valve surgery	26 (52%)
- aortic valve repair	12 (24%)
- aortic valve replacement	11 (22%)
- concomitant aortic root surgery	8 (16%)
- fibroelastoma removal	3 (6%)
Isolated ascending aorta replacement	1 (2%)
CPB time (min)	137.8 ± 47.9
Aortic cross-clamp (min)	83.1 ± 31.4
Total surgery time (min)	184.4 ± 53.2
*Postoperative management*	
Extubation in the OR	48 (96%)
PACU stay (min)	205.7 ± 69.3
ICU discharge (hours)	14.0 ± 7.4
Total days in hospital (days)	6.2 ± 2.9
In-hospital mortality	0 (%)

There were no intraoperative complications or ERAS protocol-associated events during surgery. All patients were uneventfully weaned off CPB under low dose of inotropic and vasopressor support. A total of 48 patients were successfully extubated in the OR directly after the chest closure and were transferred to the PACU. Two patients could not be extubated immediately due to insufficient spontaneous respiratory effort, but were extubated early in the PACU. None of the patients had low cardiac output after the surgery and serum lactate levels were constantly below 3.0 mmol.l^-1^. Mean serum lactate level after weaning off CPB was 1.4 ± 1.1 mmol.l^-1^.

The postoperative medication in the PACU is summarized in **Table 3**. The postoperative course in the PACU was uneventful in 44 (94%) patients. One patient required redo-surgery due to relevant bleeding after minimally invasive mitral valve repair and one patient had to be reintubated due to generalised seizure which was accompanied by global respiratory failure. This patient was subsequently found to have a minor stroke in the right frontal subcortical cerebrum potentially due to air embolism intraoperatively. The remaining third patient developed severe delirium in the PACU requiring repetitive intravenous neuroleptic drug application.

**Table 3:** Postoperative medication in the PACU.

Medication	Study cohort (n = 50)	
	Number of patients	Mean dose
Norepinephrine	9	0.027 ± 0.013 μg.kg^-1^.min^-1^
Piritramid	37	11.8 ± 6,0 mg
Oxycodon	5	6.0 ± 2.2 mg
Piritramid and Oxycodon	4	
Ondansetron	9	4.0 mg

Most patients (82%, 41/50) had an uneventful postoperative course in the general ward and could be discharged on the fifth to sixth postoperative day, according to the ERAS protocol. Postoperative complications occurred in nine patients which are displayed in the **Table 4**. Two major adverse postoperative events occurred: one patient required redo aortic valve surgery as described above. Pericardial patch detachment in the repaired unicuspid aortic valve was found during the redo surgery and was followed by aortic valve replacement using a bioprosthesis. The second patient had a generalized seizure in the PACU and had to be reintubated due to respiratory failure. Subsequent cranial CT scan revealed new subcortical hypodensities in the right frontal cerebrum, potentially due to perioperative air embolism. This patient was extubated on the third postoperative day and showed no residual neurological symptoms during the later in-hospital course.

**Table 4:** Postoperative complications. * requiring pacemaker implantation.

Event	Study cohort (n = 50)	Comment
Cardiovascular:		
- Atrial Fibrillation	6 (12%)	After AV replacement
- Permanent AV-Block III°*	2 (4%)	2 bleeding events / 1 redo
- Redo surgery	3 (6%)	due to valvular problems
Pulmonary:		
- Reintubation	1 (2%)	generalized seizure
- Nosocomial infection	2 (4%)	
Neurological:		
- Stroke	1 (2%)	1 PACU
- Delirium	2 (4%)	1 PACU, 1 general ward
General:		
- Bacteriuria	3 (6%)	
- RBC transfusion	12 (24%)	

The most common complication was postoperative atrial fibrillation which occurred in the 12% study patients. Most patients (4/6) were treated by antiarrhythmic medication only and two remaining patients underwent electrical cardioversion. Furthermore, two patients had a persisting AV Block III after aortic valve replacement in severely calcified bicuspid aortic valve stenosis and required permanent pacemaker implantation.

Physiotherapeutic ERAS protocol was followed in all 50 patients, independent from the occurrence of postoperative complications. Initial mobilization at 3 hours postoperatively was successful in all 47 patients who were extubated in the OR. Occasionally, we faced hypotension during the initial mobilization at three hours postoperatively in the PACU. No other unexpected adverse events occurred during this very early mobilization maneuver. Strict adherence to the physiotherapy protocol was well tolerated in all patients who had an uneventful postoperative course.

Full adherence to the physiotherapy protocol (i.e., from day 1 to day 4 physiotherapy units) was possible in 40/50 (80%) patients and improved significantly after the very first 20 cases. The reasons for non-adherence to physiotherapy protocol were (a) increased nausea/vomiting in 5 patients (4 of them during the early phase), (b) arrythmia (atrial fibrillation / AV block) in 3 patients (c) disabling pain in 2 patients (both in the early phase), (c) neurological events (delirium/stroke) in 2 patients, (d) redo AV surgery in 1 patient.

The adherence to preoperative and postoperative nutritional supply protocol was 100%.

As described above, PONV and disabling pain were frequent problems in the early phase of the project and limited the attachment to the physiotherapy protocol. By adjusting the protocol (e.g. by adding continuous propofol infusion to the intraoperative medication) these unrequested events could be extenuated in the late phase of the project (**Table 5**).

**Table 5:** Incidence of pain and PONV in the early vs. late phase of ERAS program.

Variable	Early phase (n = 20)	Late phase (n = 30)	p value
**Disabling pain**	6 (30%)	2 (7%)	0.03
**PONV**	7 (35%)	2 (7%)	0.02

## Discussion

*Enhanced recovery after surgery* (ERAS) protocols represent one of the key features of contemporary perioperative medicine and has been adopted by many surgical disciplines [1–5]. Establishment of ERAS based protocols demonstrated major advantages in the postoperative recovery after colorectal surgery [1–3], pancreatic surgery [6,7], urologic [9] and gynecologic surgery [10, 11]. ERAS protocols in cardiac surgery are still in their beginnings and recommendations for their establishment have been published recently in 2019. This report describes the successfull development, implementation and reevaluation of an ERAS conform protocol in minimally-invasive cardiac surgery. In contrast to most previously reported fast-track protocols limited to small-incision cardiac surgery and early extubation, our ERAS protocol includes a very comprehensive perioperative management protocol, since it addresses preoperative optimization and postoperative rehabilitation in the same way as it does for the intraoperative management. Specifically, it consists of (1) preoperative on-site consultation in an interdisciplinary team with focus on nutritional supply and optimization of the functional capacity (2) a wide-spectrum of intraoperative modifications in the CPB and anesthetic management (modified volume and temperature management, high CPB flow of 3.2 L/m2, restrictive vasopressor use) and (3) standardized postoperative rehabilitation protocol. This pilot study shows that such a protocol is feasible and safe in minimally-invasive cardiac surgery.

Historically, so-called fast-track protocols in cardiac surgery were implemented 20 years ago during the evolution phase of the modern minimally invasive heart surgery [16–18]. These early protocols addressed primarily the intraoperative phase and showed the potential for an early extubation on the ICU, shortening of the ICU stay and cost containment in the cardiac surgical setting. However, despite the further development of minimally invasive cardiac surgery and the introduction of hybrid and catheter-based cardiovascular interventions little progress has been made in the perioperative management protocols. During the last two decades, several centers have adopted fast-track postoperative management protocols worldwide, to gain beneficial effects on the internal infrastructure and costs of cardiac surgical procedures [19]. Disregarding the logistic and economic aspects of fast-track medicine, the involved health care professionals demonstrated a positive impact on the patients’ outcomes, reduction of hospital stay and associated infectious complications: e.g. pneumonia, urinary tract infection, wound infection, mediastinitis. However, integrative ERAS protocols considering the complete perioperative process have been reported only sporadically and regained an increasing interest very recently [15, 20].

The experience gained in this pilot study underlines two equally important aspects for ERAS in cardiac surgery: first, it is an integrative process consisting of the pre-, intra- and postoperative modifications, and secondly, it is strictly dependant on a team approach, similarly as it has been established for heart team in the catheter-based heart valve therapies.

Prehabilitation protocols for elective cardiac surgery are still in development [18]. The focus is on the exercise training, for example twice a week over six to ten weeks. They are considered to effectively increase patients’ functional status [19]. The WHO emphasizes the aspect of movement, which has a decisive influence on the quality of life, well-being and self-esteem of patients. Less scientific evidence is available for preoperative nutritional support. Though it is known that an adequate perioperative nutritional status is beneficial for postoperative outcome, no specific protocols for optimization of nutritional status in cardiac surgical patients have been established so far [20]. The third key feature for prehabilitation is respiratory exercizing [21]. All three components of prehabilitation have been addressed in our ERAS protocol. Allthough all of them seem quite reasonable, there is only limited evidence regarding their specific impact on the patients’ outcome. Given the heterogeneity of prehabilitation protocols in different surgical settings, previously published meta-analysis was unable to demonstrate a significant positive impact on the patients’ outcome, Nonetheless, reduced need for additional rehabilitation treatment could be demonstrated [22].

Regarding the intraoperative phase, main advancements in the elective cardiac surgery were made in regard to the cardiovascular and anesthesiological management. We strongly believe that the CPB-flow is much more important than the mean arterial pressure. Therefore, our cardiopulmonary bypass flow was targeted to 3.2 l min^-1^ in all patients. Recent study by Holmgaard et al. supports our CPB management, showing that vasopressor-induced increase in the mean arterial pressure (MAP) at constant CPB flow of 2.4 l min^-1^ has a negative effect on cerebral oxygenation values [23]. Mean arterial pressure in the low MAP group in the above-mentioned study was 45 mmHg as compared to MAP of 50 mmHg in our ERAS protocol [23]. Though there is no definite scientific evidence regarding specific cut-off value of an increased CPB flow, a restrictive vasopressor therapy under surveillance of cerebral oxygenation seems to be favorable. Maintenance of adequate end-organ perfusion during the surgery, the restoration of physiological conditions such as normothermia and administration of short-acting anaesthetics allow on-table extubation. There are different ways to perform fast-track anesthesia [24]. Our protocol did not only address on-table extubation, but also focus on the early postoperative patient comfort. Therefore, adequate pain management and nausea/vomiting prophylaxis play an essential role.

Our pilot study was able to demonstrate the feasibility and safety of ERAS protocol in the setting minimally-invasive cardiac surgery. After 3,5 hours, patients were considered fit for discharge to a low/ intermediate care unit providing continuous monitoring of ECG, pulse oximetry and intermittent non-invasive blood pressure monitoring. Due to infrastructural and logistic circumstances in the implementation phase of our program, these patients were transferred to ICU overnight. Although our study was not designed to show the superiority of ERAS protocol as compared to the standard perioperative management, perioperative morbidity was very comparable to the recent findings in patients with sutureless aortic valve replacement [25].

## Conclusion

Our ERAS protocol seems to be feasible and safe in minimally-invasive cardiac surgery and has an obvious potential to improve patients’ outcome. Prehabilitation with functional / respiratory exercises and nutritional support, intraoperative use of short-acting anaesthetics with on-table extubation and prevention of discomfort such as nausea and vomiting, in combination with an early postoperative mobilization and functional training are the key components of our protocol. Successful implementation is only possible in a multidisciplinary team approach.

## Supporting information

S1 Raw Data Data set with the collected parameters of the analysed 50 patients of the study. (CSV)
